# Lichen planus of the esophagus: a case report

**DOI:** 10.3389/fmed.2023.1233566

**Published:** 2023-12-20

**Authors:** Elvira Guarner, Fatimetu Mohamed, David Busquets, Begoña Fuertes Negro, Rosa Ortiz, Berta Oliveras, Carlos Huertas, Carme López

**Affiliations:** ^1^Department of Gastroenterology, Hospital Universitari Doctor Josep Trueta de Girona, Girona, Spain; ^2^Department of Pathology, Hospital Universitari Doctor Josep Trueta de Girona, Girona, Spain

**Keywords:** lichen planus, deglution disorders, lichen planus oral, esophageal squamous dysplasia, esophageal squamous cell carcinoma

## Abstract

Esophageal lichen planus (ELP) is an inflammatory disorder that affects the skin, cutaneous appendages and mucous membranes. The esophageal involvement is rare. We present the case of a 70-year-old woman with years of dysphagia and a history of erosive lichen planus involving the vulva, vagina, gingiva, and skin, who was eventually diagnosed with esophageal lichen planus. The patient's condition was refractory and progressed to the development of intraepithelial squamous neoplasia. We reviewed the literature on this condition.

## Introduction

Lichen planus is an inflammatory disease of uncertain etiology that affects the skin, cutaneous appendages, and mucous membranes with a prevalence of 1% in the general population. Esophageal involvement is rare. It is suggested that it is an underdiagnosed condition that could be present in up to 25–50% of patients with lichen planus, being more frequent if there is an involvement of the oral mucosa. It presents a described risk of malignancy of ~5–6%, but there is currently no clinical guideline for the treatment and follow-up of these patients. We present a case of esophageal lichen planus that did not respond to medical treatment and which eventually led to the development of diffuse squamous neoplasia.

## Case report

This is a 70-year-old woman with a history of obesity, obstructive sleep apnea syndrome, fibromyalgia, dyslipidemia, subclinical hypothyroidism, and a history of vulvar lichen planus diagnosed in 2013 (not confirmed in biopsies), but when she presented in 2015 with lichen planus lesions in the body and oral mucosa (pruritic violaceous papular lesions on the lumbosacral area and intraoral lesions on bilateral buccal mucosae with whitish reticulation), it was oriented as an erosive vulvovaginal-gingival and cutaneous lichen planus. The patient consulted in 2019, referred by primary care for gastroesophageal reflux symptoms that did not improve with antisecretory treatment. Upon reassessment, the patient explained she was experiencing heartburn, sporadic cervical impactions, and retrosternal pain.

**Figure 1:**
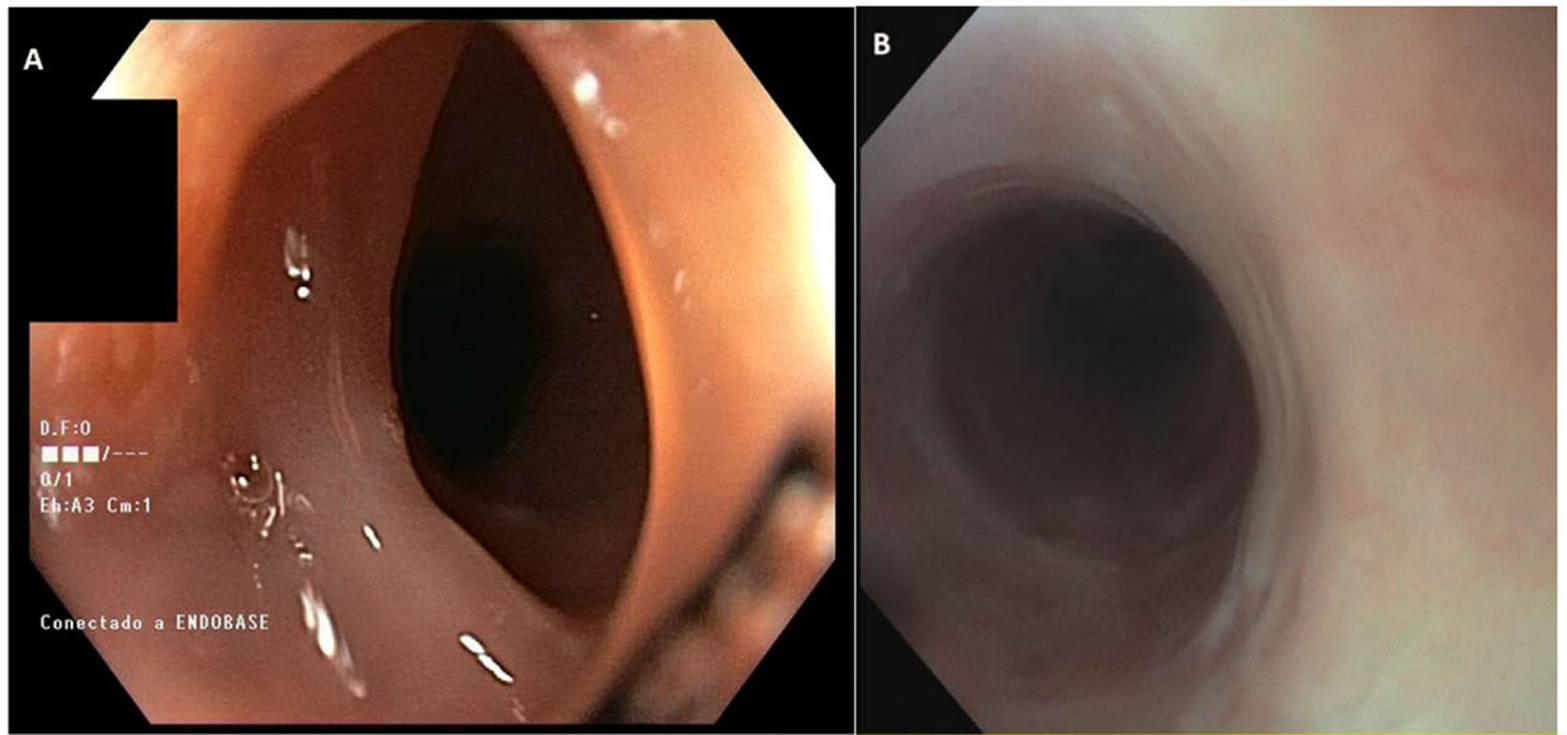
**(A)** Endoscopic image of the upper third esophageal stenosis. **(B)** Endoscopic image of the fibrous ring in the cervical esophagus.

A gastroscopy was performed in June 2019, which showed scarring changes in the middle and distal esophagus and a small hiatal hernia, oriented as reflux esophagitis. Proton pump inhibitors were prescribed, following which initial clinical improvement was observed. Due to clinical worsening with high dysphagia to solids and liquids and dysphonia, a new endoscopic study was performed in October 2020, which showed a semicircular substenosis just below the upper esophageal sphincter (Figure 1A) that did not allow the passage of a 12 mm endoscope but only a 10 mm instrument. Due to prevailing symptoms, the patient was rescheduled for dilatations. During the third endoscopy, a 12 mm pneumatic balloon dilation was performed and the rest of the esophageal mucosa was described as normal.

Despite some clinical improvement, the patient still experiences symptoms, so a second opinion was requested to rule out eosinophilic esophagitis. A new endoscopy was performed in November 2021, which described a passable cervical fibrous ring (Figure 1B) and a fibrotic distal esophagus that was biopsied, resulting in moderate dysplasia of the squamous epithelium without an increase in eosinophils. Due to the finding of dysplasia, another esophagogastroduodenoscopy was repeated in February 2022, with biopsies taken from the distal, middle, and proximal esophagus, all showing moderate dysplasia. Immunohistochemical investigations for herpes simplex virus (HSV)-I and II, cytomegalovirus (CMV), and human papillomavirus (HPV)-16, as well as periodic acid–Schiff stain (PAS) and Grocott examinations for fungal infections, turned out to be negative. The study was completed with a positron emission tomography (PET)/computed tomography (CT) scan, which showed no suggestive lesions of malignancy. The case was presented to the committee recommending endoscopic follow-up in 6 months.

**Figure 2:**
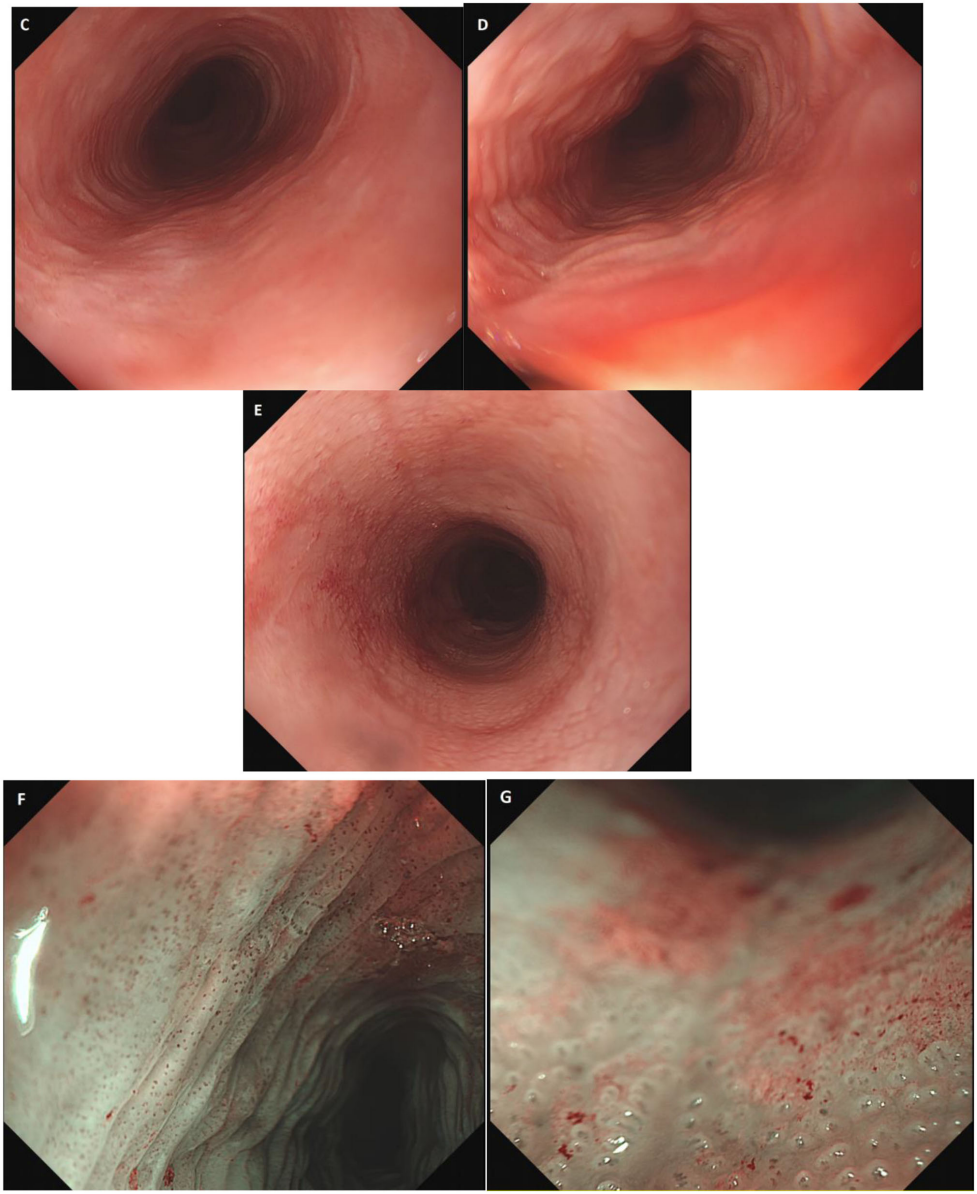
**(C–E)** Endoscopic images of esophageal rings, longitudinal stripes, and whitish network-like mucosa. **(F, G)** Endoscopic images in narrow band imaging (NBI) showing vascular changes.

**Figure 3:**
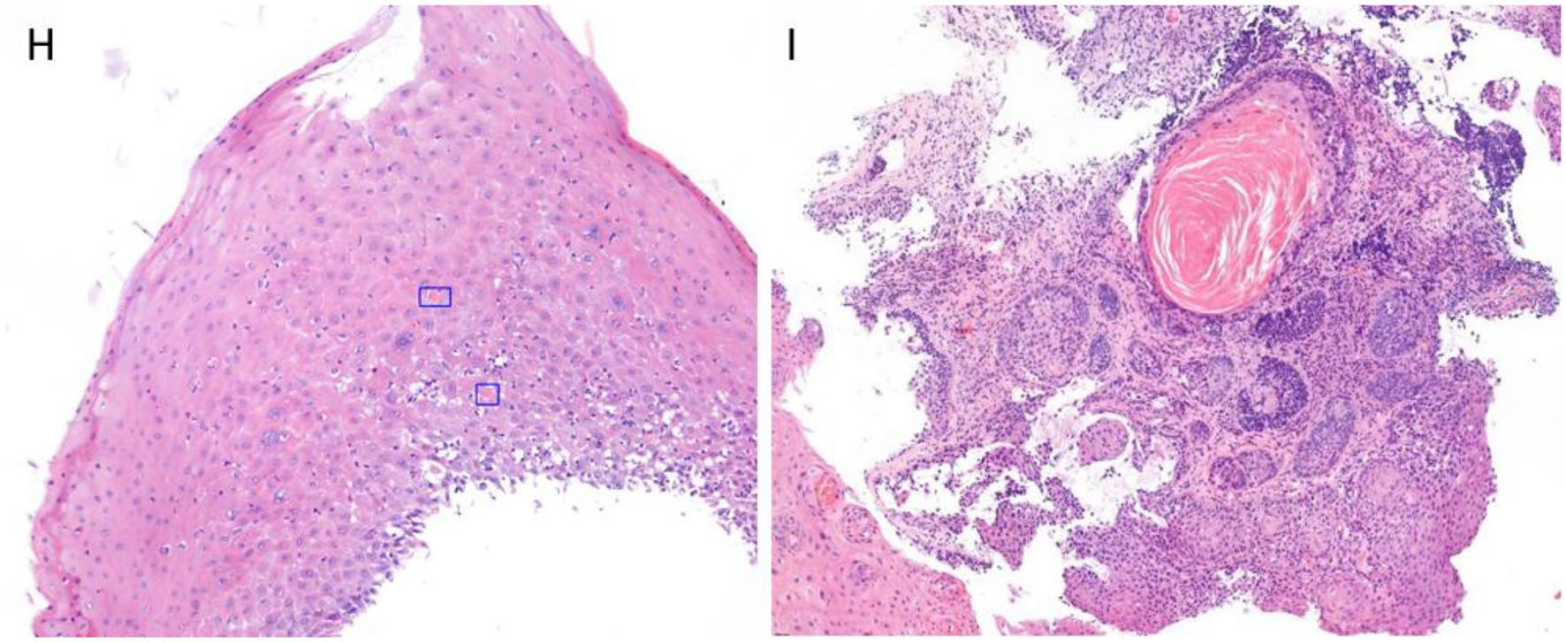
**(H)** HE × 40 staining. Endoscopic biopsy contains only the superficial squamous epithelium, showing intraepithelial lymphocytes predominantly in the lower layers, as well as “Civatte bodies” (highlighted). **(I)** HE × 20 staining. Esophageal squamous dysplasia, high grade with isolated focus, probably representing dysplastic involvement of submucosa gland ducts.

In subsequent endoscopic examinations (August and November 2022), previously unreported esophageal changes were observed, such as a decrease in lumen caliber, generalized trachealized rings (Figure 2C), longitudinal striations (Figure 2D), a new stenosis at 18 cm from the dental arch that is endoscopically dilated, erosions and friability, and a white “reticular-like” or atrophic-looking mucosa (Figure 2E), with abnormal vascular changes and pinpoint vessels along the entire esophageal path (Figures 2F, G). After a thorough review of the patient's medical history, esophageal lichen planus was suspected. Multiple biopsies at different levels were obtained, which showed esophageal mucosa with hyperplasia of the superficial epithelium, lymphocytic infiltrate predominantly in the lower layers, and a hydropic degeneration of basal cells. The presence of occasional dyskeratinocytes or apoptotic eosinophilic bodies (known as “Civatte bodies”) were also noted (Figure 3H). All of these were compatible with lichenoid esophagitis. Moreover, high-grade dysplasia was observed in some areas, showing both cytological and architectural atypia, which is defined by almost full-thickness epithelial disorganization with lack of maturation. Cytological atypia includes loss of nuclear polarity, marked nuclear enlargement, irregular nuclear contours, pleomorphism, hyperchromasia, and an increased number of mitoses.

**Figure 4:**
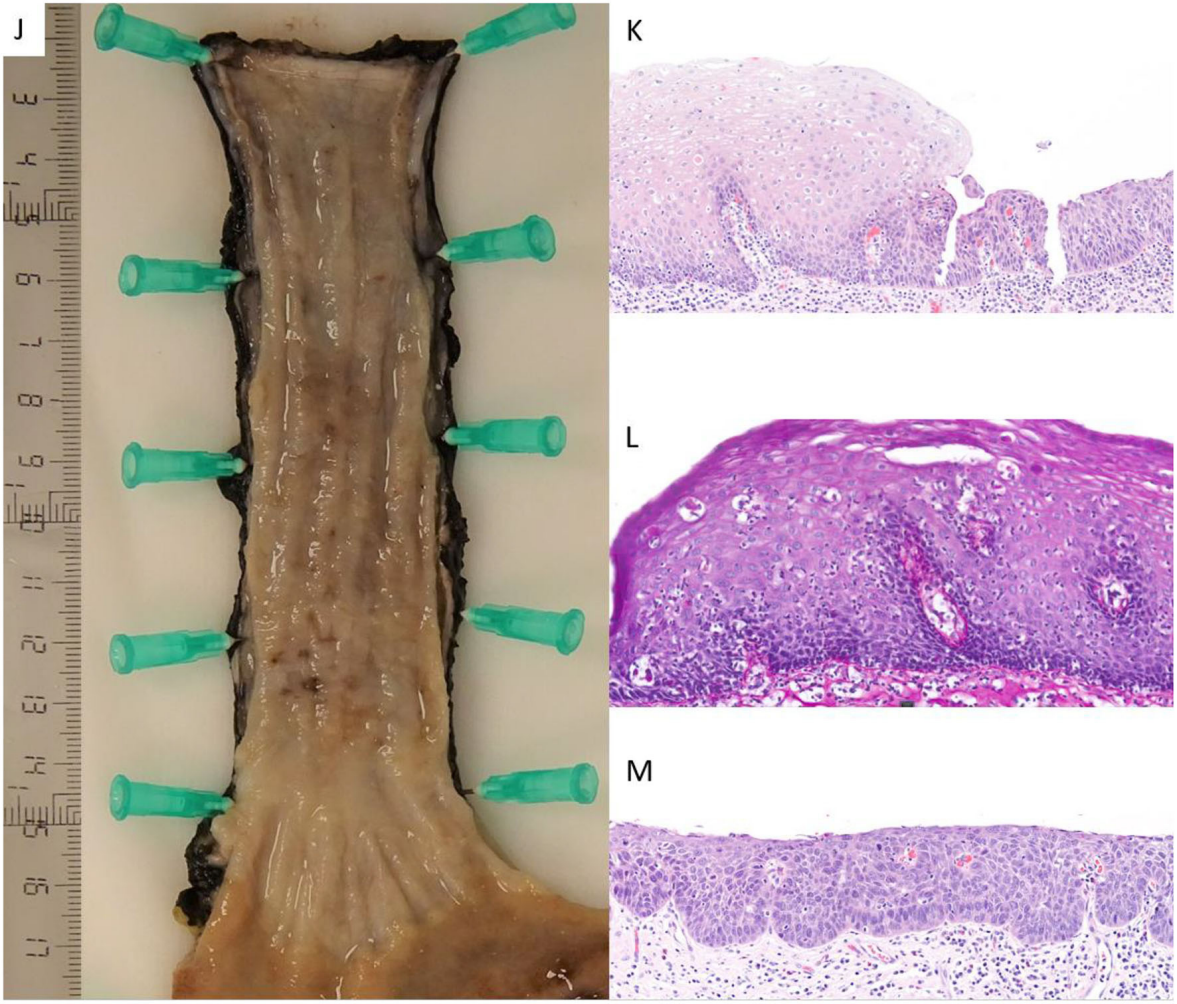
**(J)** A macroscopic picture of the surgical specimen, which shows the esophagus and the gastroesophageal union. The whole esophageal mucosa shows an irregular surface, with violaceous areas alternating with paler zones, as well as occasional punctate depressions corresponding to superficial erosions. **(K)** HE × 40 staining. Section from the surgical specimen showing esophageal mucosa with abrupt transition from lichenoid changes to frank dysplasia **(L)** PAS-diastase x55 staining. This complementary technique highlights the eosinophilic “Civatte bodies” at high magnification, in addition to hydropic degeneration of the basal cells. **(M)** HE × 40 staining. View of high-grade dysplasia with an increased number of mitoses, some of them atypical and located in upper layers of the epithelium.

The patient was prescribed oral fluticasone 0.5 mg/12 h for 3 months as described in the literature, without symptomatic improvement. A new gastroscopy was performed (February 2023) and biopsies from the middle and distal esophagus showed intraepithelial squamous neoplasia (high-grade dysplasia, Figure 3I). Given the findings and in accordance with the patient's condition, she was referred for total esophagectomy through robotic surgery (July 2023), which led to complications, including suture dehiscence with pleural and tracheal communication, as well as a hemothorax, necessitating a surgical re-intervention. She is currently hospitalized with a cervical prosthesis. The pathology report of the surgical specimen indicates high-grade dysplasia on a focally ulcerated lichenoid esophagitis without signs of invasion (Figures 4J–M).

## Discussion

Lichen planus is an inflammatory disorder of uncertain etiology that affects the skin, cutaneous appendages, and mucous membranes (1), with a prevalence of 1% in the general population (2). It more commonly affects women between the ages of 40 and 50 (3). Esophageal involvement is rare, occurring more frequently in women with dysphagia due to proximal stenosis [with the most typical involvement being proximal (3)]. Of these cases, proximal stenosis is present in up to 41% of the cases (4). It is suggested that this is an underdiagnosed condition (asymptomatic) and could be present in up to 25–50% of patients with lichen planus (5, 6), with a higher frequency if there is oral involvement (up to 89% of erosive lichen planus (ELP) cases have oral involvement) (3–5). Although endoscopic screening is not recommended in these patients, a complete anamnesis of esophageal symptoms is recommended in patients with oral lichen planus (7).

The association of ELP with other organ involvement is not entirely clear. Eisen D described they found patients with ELP having at least one other form of involvement than oral involvement (cutaneous, genital, scalp, nail and/or ocular) (7). Another study reports that among patients with ELP, 89% have oral involvement, 42% have ano-genital involvement, and 38% have cutaneous involvement (3). Similarly, in a case series of patients with ELP (4), 74% had oral involvement and 52% of women had vulvo-vaginal involvement. In this series, in 48% of the cases, the disease was initiated with esophageal involvement. Therefore, it appears necessary to conduct a comprehensive assessment of the patient when ELP is diagnosed.

Symptoms include dysphagia, odynophagia, heartburn, weight loss, and retrosternal pain, among others. The differential diagnosis includes reflux esophagitis, eosinophilic esophagitis, pill esophagitis, and primarily viral and fungal infections. Although endoscopic images are suggestive of this entity (white “mesh-like” papules, esophageal membranes, pseudomembranes, erosions, desquamation, stenosis) (6, 8), its low frequency, non-specific symptoms, and possible lack of experience of the endoscopist and pathologist can lead to delayed diagnosis or even confusion with reflux esophagitis due to desquamative involvement, requiring multiple endoscopic studies. In this case, the presence of oral and vulvar lichen planus should have raised early suspicion of this condition.

Focusing on skin involvement, lichen planus has been classically described as flat, polygonal, red-violet papules with a whitish reticulated surface (Wickham's striae) and intense pruritus, typically located in flexural areas, the lumbosacral region, and flanks (1). Up to 60–70% of cases exhibit concurrent oral and/or genital lesions (whitish reticular lesions that may be erosive). The differential diagnosis for these skin lesions is quite diverse, ranging from lupus erythematosus, guttate psoriasis, or tinea corporis, to candidiasis, leukoplakia, or mechanical agents in the oral mucosa. Histology, combined with the patient's symptoms, contributes to the definitive diagnosis (9).

The risk of malignancy is not negligible, with dysplasia or esophageal squamous cell carcinoma occurring in two reviews in 5.55 (3) and 6.1% (10) of cases, respectively. Therefore, some authors recommend annual endoscopic follow-up (10), with total esophagectomy being the treatment of choice when malignancy is present (11). There is no standardized clinical guideline, but medical treatment includes systemic and topical steroids, cyclosporine, azathioprine, and retinoids, complemented by endoscopic treatment if needed (dilation) but not systematically due to the Koebner phenomenon. Ynson et al. recommend as initial therapy 220 mcg/12 h of swallowed fluticasone propionate for 6 weeks, presenting a case (8) with a good clinical response and fewer side effects than systemic steroids. Bobadilla et al. present a case (12) with a good response to oral cyclosporine 3 mg/kg/day, with resolution of symptoms at 2 weeks and endoscopic remission at 15 weeks, without side effects. This treatment was chosen because the patient's vaginal and oral lesions did not respond previously to topical or systemic steroids.

In conclusion, this is a difficult condition to recognize for both the endoscopist and pathologist, which should be suspected in middle-aged women with refractory and high esophageal strictures and, above all, if there is a history of lichen planus in another location. Currently, there is no standardized clinical guideline for the management of these patients, so future studies should be conducted to establish an appropriate clinical approach.

## Data availability statement

The original contributions presented in the study are included in the article/supplementary material, further inquiries can be directed to the corresponding author.

## Ethics statement

Written informed consent was obtained from the patient for the publication of this case report.

## Author contributions

FM followed up on this patient during consultations. FM, DB, CH, EG, and BO performed the various gastroscopies. BN and RO contributed to the anatomopathological diagnosis. EG wrote the initial draft of the manuscript, which was reviewed by all authors. CL as the head of the department, edited the initial draft of the manuscript. All authors contributed to the article and approved the submitted version.
